# The Choice of Bread: The Association between Consumers’ Awareness of Dietary Fiber and Declared Intentions to Eat

**DOI:** 10.3390/nu12020360

**Published:** 2020-01-30

**Authors:** Marzena Jezewska-Zychowicz, Maria Królak

**Affiliations:** Department of Food Market and Consumer Research, Institute of Human Nutrition Sciences, Warsaw University of Life Sciences-SGGW, Nowoursynowska 159c, 02-776 Warsaw, Poland; marzena_jezewska_zychowicz@sggw.pl

**Keywords:** dietary fiber, fiber awareness, bread, choice of bread

## Abstract

The objective of the study was to find whether consumers declare an intention to eat bread enriched with fiber in the situation of availability of a plain bread and plain bread with grains, and how these intentions are related to their awareness of fiber in bread. The data were collected in a cross-sectional survey among 1014 Polish adults. Based on three pictures of rolls: plain wheat roll (CR), roll enriched with 12% fiber (RF), and roll topped with sunflower seeds (RSS), the participants’ perception regarding fiber content and its impact on intentions to eat were assessed. The respondents were not informed about the differences in composition of the rolls. Although RSS contained only slightly more fiber (0.98 g) than CR (0.81 g), and much less than RF (3.08 g), most of the respondents indicated RSS as containing the most fiber (50.8%) and declared their intention to eat it (39.0%). Respondents who pointed out the importance of fiber, and grains or wholemeal flour addition when making purchase decision, were more likely to declare an intention to eat RSS compared with CR. The low interest in fiber content in a diet increased the chances of declaring an intention to eat CR and RF. People less educated and with low incomes were more likely to declare an intention to eat CR rather than RSS. While people from rural areas were more likely to choose RSS compared with CR and RF. In conclusion, there is interest in bread enriched with fiber, but consumers experience difficulties in recognizing it. Declared intentions to eat each rolls were determined to the greatest extent by the perception of the roll as source of fiber. Thus, educational activities should be focused on consumers’ perception of fiber-rich products and their skills related to the selection of high-fiber foods.

## 1. Introduction

Despite ongoing efforts to promote adequate eating behavior and increase fiber intake, average fiber consumption still remains lower than the recommended daily amount [1,2,3]. This is mainly due to too low vegetable and fruit and wholemeal product consumption [4,5]. Although much is already known about the reason behind insufficient fiber consumption [6,7,8,9], there are still some unexplained motives and ambiguous studies. Previous research has presented conflicting results related to associations between fiber intake and fiber awareness. According to some studies, low intake of whole grains results mainly from a lack of knowledge about their beneficial effects on health [10,11,12]. However, other studies indicate high knowledge about dietary fiber and consumers’ ability to link fiber intake to its preventive effects on the body function [13,14]. Consumers are aware of the beneficial influence of dietary fiber and wholegrain products on their health [14,15]; they can also identify products that are good sources of fiber [2,16]. 

The discrepancies found in the results may be explained through reference to other studies indicating that many factors can limit the effect of fiber awareness on fiber intake, including the belief of high-fiber food as unpalatable [10], too many competitive products on the market, higher price of wholemeal products in comparison with their refined equivalents, and unwillingness to change existing habits [8,9,17]. The availability of high-fiber foods, as well as the relationship between fiber awareness and the ability to make an adequate choice when selecting food seems to be very important in explaining and predicting fiber intake. Some previous studies have shown that consumers recognize products that are rich in fiber, and those with whole grains [13,18]. However, the question remains, whether consumers can recognize the addition of fiber to an already known product.

The consumption of fiber below the recommended amount encourages the development of new products with increased fiber content [19,20]. The acceptance of these products by consumers is diversified and mainly determined by the product in which the modification was made [21,22,23,24]. Therefore, the choice of carrier of added fiber may be decisive with regard to the acceptance of a new product. Moreover, product modification may affect its sensory properties, which may be crucial in its acceptance or rejection [10,25].

The appearance of products with added fiber may only slightly differ from that of their counterparts without such an addition. Therefore, bakery products with added fiber, especially in a micronized form, may not be perceived by the consumer as products with an increased amount of fiber. The sale of bread and rolls without packaging does not give one the opportunity to be informed about the change in the composition of products. At the same time, such sales of bread are still common not only in Poland, but also in other countries. Because the results of previous studies have shown positive association between information on fiber addition on the label and the acceptance or the willingness to purchase bread with fiber [22,26,27], labels seem to be an easy, accessible, and effective tool to inform about the change.

Although some studies are focused on the assessment of consumers’ acceptance of the attributes of various products with added fiber [23,28], there are no studies focusing on consumers’ interest in choosing such products when other substitute products are available (among others, products that are not a good source of fiber and can be seen as such, e.g., stained with caramel, and those with a negligible addition of fiber, e.g., rolls topped with sunflower seeds) or no information about the addition of fiber has been provided. Thus, the objective of this study was to answer the question whether the consumers declare an intention to choose bread enriched with fiber in the situation of availability of plain bread and plain bread with sunflower seeds, and also how these intentions are related to their interest in dietary fiber.

## 2. Materials and Methods

### 2.1. Study Sample

Data were collected in October 2014 through a cross-sectional survey within project ‘Bioproducts, innovative technologies of pro-health bakery products and pasta with reduced caloric value’ [29]. According to the study design, recruitment and data collection were conducted by a research agency—BIOSTAT Group—in accordance with the ESOMAR (European Society for Opinion and Marketing Research) code of conduct using the CAPI (Computer-Assisted Personal Interview) technique. The survey was conducted in each of the sixteen voivodeships in Poland. After selecting the starting addresses, the ‘random route’ method was used [30]. The study sample included people aged 21 or more who met following recruitment criteria, i.e., (1) being responsible for food purchases or making cooperative food purchases within the household (*Who usually makes food purchases in your household?: (1) Only me; (2) I make most of the food purchases; (3) I sometimes make food purchases and sometimes somebody else does; (4) Somebody else makes most of the food purchases; (5) I never make food purchases*), and (2) eating at least two slices of bread a day *(How often do you eat bread?: (1) I eat 2 or more slices of bread several times during the day; (2) I eat bread one time during the day, however at least 2 slices of bread; (3) I eat less than 2 slices of bread during the day*). Respondents who answered that they did not participate in the purchasing of food (*Somebody else makes most of the food purchases or I never make food purchases*) and that they ate less than two slices of bread a day (*I eat less than 2 slices of bread during the day*) were excluded from the study. Details concerning the sociodemographic characteristics of the study sample are displayed in Table 1.

### 2.2. Design of the Study

The study consisted of three parts. In the first part, participants watched pictures of three different rolls: a plain roll made from white wheat flour (control roll—CR), a plain roll from white wheat flour enriched with 12% fiber after a micronization process (RF), and a plain roll from white wheat flour topped with sunflower seeds (RSS). Each roll was presented as a whole roll (uncut) and after being cut into 2 parts with visible crumb and color. Rolls were prepared in the Laboratory of Technologies, Faculty of Human Nutrition and Consumer Sciences. The rolls did not differ in size (50 g) and color with two exceptions: The roll with fiber addition had a slightly darker color compared to the control roll due to the addition of fiber, and the roll topped with sunflower seeds differed from the plain roll with respect to the presence of seeds on the surface. In addition, the rolls differed in the amount of fiber, namely, RF contained 3.08 g of fiber, CR—0.81 g of fiber, and RSS—0.98 g of fiber. In this part of the study, the respondents were asked to choose the roll they would consume most willingly (an intention to eat).

In the second part of the study, participants answered the questions that concerned issues such as the importance of selected food choice motives, checking the content of fiber on the food label, interest in fiber content in a diet, and perception of rolls as a fiber source.

In the third part of the study, participants watched pictures of the three tested rolls once again in order to choose the roll which in their opinion has the largest amount of fiber.

### 2.3. Questionnaire 

The questionnaire included questions concerning following issues:

1. Intention to eat—participants were asked to point out which one of the three rolls presented, i.e., CR, RF, and RSS, they prefer to eat. Question: *Which of the presented rolls would you like to eat?*
2. Perceived importance of grain addition and/or the use of wholegrain flour, and fiber addition, when making a purchasing decision—Question: *How important for you are the following attributes of bread when making the decision to buy? Please rank the listed attributes from the most important (1) to the least important (10); attributes: taste, freshness, appearance, naturalness, health benefits, addition of grains, fiber addition, familiarity with the product, attractive price and shelf life*.
3. Interest in fiber content in a diet—Question: *Are you interested in the amount of fiber in your diet?* The answers were based on a 5-point scale: 1—I am interested; 2—I am rather interested, 3—neither interested nor uninterested, 4—I am not very interested; 5—I am not interested.
4. Checking the content of fiber on food labels was measured with the question: *Do you check the content of fiber in a food product when you make purchases?* The answers were based on a 5-point scale described as 1—yes; 2—rather yes; 3—neither yes nor no; 4—not really; 5—no.

Perception of roll as a source of fiber—participants were asked to point out the roll containing the most fiber. Question: *Which of the presented rolls contains the most fiber?* They were asked to point out one roll among the three tested rolls: CR, RF, and RSS.

The questionnaire collected information about sociodemographic characteristics of the study sample as well, including gender, age, education, place of residence, and self-assessed income (Table 1).

### 2.4. Statistical Analysis

Descriptive statistics including frequency distributions were carried out. Opinions on the importance of the addition of grain and/or use of wholegrain flour and the importance of fiber addition were separated into three categories based on tertile distribution: important (1st tertile), of limited importance (2nd tertile), and unimportant (3rd tertile). 

Associations between sociodemographics, perceived importance of grain addition and/or the use of wholegrain flour, and fiber addition, when making a purchasing decision, interest in fiber content in a diet, checking the content of fiber on food labels, perception of roll as a source of fiber, and intention to eat rolls were verified using logistic regression analysis. Sociodemographic variables were: gender—female (ref. male); education—lower than secondary education and secondary education (ref. higher education); place of residence—rural and city with less than 100,000 citizens (ref. city with more than 100,000 citizens), and self-assessed income—low and average (ref. high income).

Other variables included in logistic regression analyses were as follows: 

Grain addition and/or the use of wholegrain flour—an important attribute—1st tertile, and an attribute of limited importance—2nd tertile (ref. an unimportant attribute—3rd tertile); 

Fiber addition—an important attribute—1st tertile, and an attribute of limited importance—2nd tertile (ref. an unimportant attribute—3rd tertile); 

Low interest in fiber content in a diet—scores 3, 4, and 5 from the 5-point scale (ref. high interest—scores 1 and 2 from the 5-point scale); 

Checking the content of fiber on food labels—scores 1 and 2 from the 5-point scale (ref. not checking—scores 3, 4, and 5 from the 5-point scale). 

Perception of roll as a source of fiber—score 1 for a roll considered as containing the most fiber (ref. other rolls separately). 

Odds ratios (ORs) represented the chances of adherence to those who declared an intention to eat each roll (CR, RF, and RSS), separately. Wald’s test was used to assess the significance of ORs. *p*-value < 0.05 was considered as significant for all tests. 

Statistical analysis was conducted using IBM SPSS Statistics for Windows, version 24.0 (IBM Corp, Armonk, NY, USA).

## 3. Results

### 3.1. Characteristics of the Study Sample with Regard to Fiber-Related Variables

The respondents’ opinions on fiber-related variables are presented in Table 2. 

The importance of the attributes ordered based on their role in making the decision regarding bread purchase was defined as follows: freshness (mean value 3.3; standard deviation 2.1), taste (3.7; 2.7) naturalness (4.8; 2.4), health benefits (5.4; 2.9), price (5.4; 2.9), shelf life (6.2; 2.6), familiarity with the product (6.4; 2.5), appearance (6.4; 2.4), grain addition, and/or the use of wholegrain flour (6.4; 2.8) and fiber addition (7.0; 2.6). 

Although use of grain and/or wholegrain flour and fiber addition were ranked as 9th and 10th, standard deviations indicate a relatively large dispersion of opinions on the importance of these attributes among the participants of the study. The tertile distribution of the opinions regarding the importance of both attributes in making bread purchase decisions is presented in Table 2.

Only 40.9% of participants declared their interest in fiber content in a diet. RF was indicated as the one containing the most fiber by 31.7% of the sample. However, most of the respondents indicated RSS as containing the most fiber. An intention to eat this roll was declared by 39.0% of respondents, while an intention to eat RF was declared by 34.2% of the study sample (Table 2).

### 3.2. Sociodemographic Variables as Predictors of Intention to Eat Rolls

Participants with an education lower than secondary compared to those with higher education and people who declared low income in comparison with those with high income were more likely to declare an intention to eat CR compared to RSS. Participants from rural areas as opposed to those from cities with more than 100,000 citizens were less likely to declare an intention to eat CR when compared to RSS (Table 3).

Participants with an education lower than secondary compared to those with higher education and people declaring a low income in comparison with those with a high income were less likely to declare an intention to eat RSS when compared to RF. However, people from rural areas in comparison with those from large cities were more likely to declare an intention to eat RSS when compared to the other rolls (Table 3). 

Participants living in rural areas compared to those from cities with more than 100,000 inhabitants were less likely to declare an intention to eat RF when compared to RSS (Table 3).

### 3.3. Fiber-Related Variables as Predictors of Intention to Eat Rolls

Participants who declared their intentions to eat CR were over ten times more likely to perceive it as containing the most fiber compared to those who declared an intention to eat RF. Moreover, they were almost four times more likely to perceive it as containing the most fiber compared to those who declared an intention to eat RSS. People declaring a low interest in fiber content in a diet in comparison with those declaring a high interest were more likely to declare an intention to eat CR. Respondents who declared the importance of fiber addition and use of grains or wholemeal flour during the selection of bread were less likely to declare an intention to eat CR. These relationships were observed only when the declared intentions to eat CR and RSS were compared. No associations were observed when the intentions to eat CR and RF were compared (Table 3).

Participants who declared their intention to eat RSS were over seven times more likely to perceive it as containing the most fiber compared to those who declared an intention to eat RF, and four and a half times more likely compared to those declaring an intention to eat CR. People declaring low interest in fiber content in a diet were less likely to point out RSS as their preferred choice for consumption. People declaring the importance of the addition of fiber and grains or wholemeal flour during the selection of bread were more likely to declare an intention to eat RSS. These relationships were observed only when the declared intentions to eat RSS were compared to the declarations concerning CR (Table 3).

Participants who declared an intention to eat RF were less likely to perceive it as containing the most fiber in comparison with CR and RSS. People declaring the importance of the addition of grains or wholemeal flour during the selection of bread were less likely to declare an intention to eat RF. People declaring low interest in fiber content in a diet were more likely to declare an intention to eat this roll when compared to RSS (Table 3).

## 4. Discussion

Rolls evaluated in the study were similar in terms of composition, i.e., they were prepared according to the recipe used for the production of plain wheat rolls. The only difference was the varied amount of fiber in the roll. Consumers evaluated the plain wheat rolls (CR), the roll enriched with 12% fiber after the micronization process (RF), and the roll topped before baking with sunflower seeds (RSS). RSS contained only slightly more fiber (0.98 g) than CR (0.81 g), but topping it with sunflower seeds may have suggested that the product, due to the presence of grains, contains more fiber. Such a selection of rolls allowed finding out whether the appearance of the product influenced the study participants’ opinions on the fiber content and their intentions to eat such a product. Half of the respondents (50.8%) chose RSS as the best source of fiber. Thus, the grains are recognizable as a source of fiber [2,18], but a negligible addition of sunflower grains in our study was insufficient to rate the roll as a good source of fiber. Among the remaining respondents, twice as many of them indicated RF as a good source of fiber compared to CR. This finding signifies that the color of the product may provide information about the fiber content. A darker color of the bread may result from the use of flour from high milling. However, for some consumers, it is difficult to distinguish wholegrain products with high fiber content due to the variable composition of such breads [2,31]. On the other hand, the selection of a plain roll as containing the most fiber is an unexpected result. Such a belief is particularly threatening, with intensive educational activities emphasizing the importance of fiber in disease prevention [32]. It can justify incorrect behavior of consumers associated with frequent consumption of plain wheat bread and can also undermine control over food intake when it is perceived as healthy [33,34].

The perception of the roll as a good source of fiber showed the strongest relationship with the declared intention to eat. Consumers were more likely to declare an intention to eat RSS when it was perceived as a good source of fiber, when the reference roll was CR and RF. That confirms the relationship between knowledge and choice of cereal products demonstrated in previous studies [35,36,37].

However, there are still consumers with lack of knowledge about fiber and its source [11,12]. In the study, the likelihood of choosing CR when it was perceived as a good source of fiber was 10 times higher compared to RF. Probably, a slight difference in their appearance could affect the expectations toward sensory attributes. The belief among consumers that darker bakery products are unpalatable [10] and a lack of understanding about their origin could strongly influence the declared intentions of their consumption. 

In our study, only slightly more participants declared an intention to eat the roll topped with sunflower seeds than the roll enriched with fiber (39.0% vs. 34.2%). This may be due to their incorrect knowledge of fiber sources, but also to the lack of information about added fiber. Not being familiar with the addition of fiber to the plain wheat roll, participants could choose this roll because of its color indicating a higher flour milling. One quarter of people chose CR, which may be the result of both their previous eating habits and their preferences [26,38], but also of not paying attention to fiber in their daily diet or believing they meet their fiber needs [39].

Our research showed that people declaring low interest in fiber content in a diet were more likely to declare an intention to eat CR and RF compared to an intention to eat RSS. Thus, the presence of seeds on the surface of the roll did not encourage them to consume such a roll. There were no differences in the declared intentions to eat CR and RF after taking into account the interest in fiber content in a diet, which may confirm the lack of ability to associate the darker color of the roll with fiber.

Participants’ interest in fiber content in a diet and importance of motives in bread choice were associated with a lower probability of declaring an intention to eat CR, but only when CR was compared with RSS. Inverse associations with an intention to eat RSS were observed, i.e., interest in fiber content in a diet and importance of motives in bread choice increased the chances of declaring an intention to eat RSS. This finding may confirm an incorrect belief that rolls topped with sunflower seeds are a good fiber source; however, this was not defined in the study.

Information about the addition of fiber on the label is therefore necessary to inform consumers about the change in formulation. However, the results of the study did not confirm the relationship between declared intention to check the content of fiber on food labels and the intention to eat the rolls. This requires further research, in which the selection of rolls will also include information about the content of fiber. Some previous studies have shown that the provided information about the addition of fiber had an impact on the declared choice of product [26,40,41]. In other research, such information did not show any relationship with the acceptance of a product, probably because of perceiving it already as being high in fiber [18].

None of the fiber variables showed any relationship with the declared intention to eat RF when it was compared with CR. It can be assumed that this may relate to the lack of skills in associating the darker color of the roll with fiber addition. CR was perceived as containing less fiber due to the well-known attributes of plain wheat flour, while RSS as containing more fiber due to the presence of grains. This assumption is confirmed by the relationship between the perception of the roll as containing the highest amount of fiber and the intention to consume it. If the roll was perceived as containing the most fiber, the chances of declaring intentions to eat it increased. This relationship did not apply to RF, which may result from the lack of experience associated with this type of bread and difficulties in interpreting the darker color, and at the same time indicates the usefulness of labels with information about added fiber. However, in our study, only 18.3% of respondents declared using labels to get information about fiber content. In other studies, interest in information on the label is usually greater [14,26,42]. In further research, it is necessary to include not only the consumers’ interest in labels but also competences associated with using them when making a purchase decision.

Some studies have suggested that demographic variables, such as gender, age, and education, are associated with nutritional behaviors [15,21,43,44]. This was also reflected in our sample. People from rural areas were less likely to declare an intention to eat RF and more likely to choose RSS than residents of large cities, while people with low incomes were more likely to choose CR and less likely to choose RSS compared to those with high incomes. However, no differences in intentions to eat were observed in gender groups. In previous studies, differences between men and women regarding their knowledge about nutrition as well as nutritional behaviors have been reported [11,36,45,46], which is mainly explained by the greater involvement of women in providing food for the family [21,47], as well as attaching greater importance to their own appearance [48].

The strength of our results is the relatively large sample from the Polish population. Nevertheless, the findings have some limitations. The sample included only those solely or jointly responsible for the family’s grocery shopping. The cross-sectional design of the study and data collection at a single point in time did not allow for conclusions to be drawn about causality, but only about the associations between variables. Moreover, the assumption that the sunflower seeds on the surface of the roll would be treated by respondents as a source of dietary fiber, similarly to grains attached, limited our results. Moreover, the real environment in which the selection of bread takes place and the use of the products themselves rather than product pictures would provide a better reflection of the conditions under which purchasing decisions are made. However, this was not logistically or economically possible in this study, considering the size of the sample. Due to the abovementioned limitations, our findings should not be generalized to populations with different cultural backgrounds. However, we hope the study provides an interesting insight into determinants of bread choice.

## 5. Conclusions

Our findings showed that only one third of the study sample declared an intention to eat rolls enriched in fiber. Thus, the addition of fiber in the natural form (sunflower seeds) proved more convincing to consumers than a micronized fiber enrichment even if the grains used contained less fiber. Respondents who declared the importance of fiber, and grains or wholemeal flour addition, during the selection of bread were more likely to declare an intention to eat the roll topped with sunflower seeds than other rolls.

Decisions regarding the choice of rolls for consumption reflected the limited application of fiber information on food labels, whereas skills to recognize the source of dietary fiber proved to be more important when declaring their intention to eat a roll. Thus, our results indicate the need to develop educational programs to improve skills related to the selection of high-fiber foods and to strengthen the importance of motives concerning fiber in cereal product choice. The results relating to the roll enriched with fiber indicate that packaging with information about fiber content may be of great importance to increase consumers’ awareness concerning bread.

## Figures and Tables

**Table 1:** Study sample characteristics.

Sociodemographic Characteristics		Total Sample	
		*N* = 1014	%
Gender	Female	552	54.4
	Male	462	45.6
Age (years)	Mean; SD	42.1; 13.1	
Education	Lower than secondary	294	29.0
	Secondary	465	45.9
	Higher	255	25.1
Place of residence	Rural area	373	36.8
	City > 100,000 residents	327	32.2
	City ≤ 100,000 residents	314	31.0
Self-assessed income	Low (‘insufficient’ or ‘sufficient only for basic needs’)	202	19.9
	Average (‘we can afford some, but not all expenses’)	578	57.0
	High (‘sufficient’)	234	23.1

*N*—number of participants; SD—standard deviation.

**Table 2:** Respondents’ opinions and intention to eat rolls.

Variables	%	Mean	SD	Range ^a,b^
**Intention to eat**				
Plain roll (CR)	26.8			
Plain roll topped with sunflower seeds (RSS)	39.0			
Plain roll enriched with fiber (RF)	34.2			
**Perceived importance of grain addition and/or use of wholegrain flour ^a^**		6.4	2.8	1–10
Important attribute (1st tertile)	35.7	3.1	1.4	1–5
Attribute of limited importance (2nd tertile)	31.3	7.2	0.8	6–8
Unimportant attribute (3rd tertile)	32.0	9.4	0.5	9–10
**Perceived importance of fiber addition ^a^**		7.0	2.6	1–10
Important attribute (1st tertile)	37.5	4.1	1.6	1–6
Attribute of limited importance (2nd tertile)	24.1	7.6	0.5	7–8
Unimportant attribute (3rd tertile)	38.5	9.5	0.5	9–10
**Interest in fiber content in a diet ^b^**		2.9	1.2	1–5
Low (scores 3, 4, 5)	59.1	3.7	0.8	3–5
High (scores 1, 2)	40.9	1.6	0.5	1–2
**Perception of roll as a source of fiber**				
CR as containing the most fiber	17.6			
RSS as containing the most fiber	50.8			
RF as containing the most fiber	31.7			
**Checking the content of fiber on food labels ^b^**		3.7	1.2	1–5
Checking (scores 1, 2)	18.3	1.8	0.4	1–2
Not checking (scores 3, 4, 5)	81.7	4.2	0.8	3–4

^a^ 1—the most important; 10—the least important; ^b^ 1—yes; 2—rather yes; 3—neither yes nor no; 4 —not really; 5—no.

**Table 3:** Adjusted associations between intention to eat a roll, fiber-related variables, and sociodemographic characteristics (Adjusted Odds Ratios with 95% Confidence Intervals).

Variables	Choice of Plain Roll (CR)		Choice of Plain Roll Enriched with Fiber (RF)		Choice of Plain Roll Topped with Sunflower Seeds (RSS)	
	Ref. RF	Ref. RSS	Ref. CR	Ref. RSS	Ref. CR	Ref. RF
	e^β^ (95% Cl)	e^β^ (95% Cl)	e^β^ (95% Cl)	e^β^ (95% Cl)	e^β^ (95% Cl)	e^β^ (95% Cl)
**Gender** (*ref. male*)						
Female	0.87(0.59; 1.26)	1.46 (0.97; 2.18)	1.16 (0.79; 1.69)	1.38 (0.97; 1.95)	0.69 (0.46; 1.03)	0.73 (0.52; 1.03)
**Education** (*ref. higher*)						
Lower than secondary education	1.44 (0.84; 2.45)	2.15 ** (1.22; 3.79)	0.70 (0.41; 1.18)	1.19 (0.74; 1.92)	0.46 ** (0.26; 0.82)	0.84 (0.52; 1.35)
Secondary education	1.18 (0.73; 1.93)	1.57 (0.94; 2.63)	0.84 (0.52; 1.38)	1.07 (0.71; 1.59)	0.64 (0.38; 1.06)	0.94 (0.63; 1.40)
**Place of residence** (*ref. city with more than 100,000 citizens*)						
Rural	1.05 0.66;1.69)	0.58 * (0.35; 0.95)	0.95 (0.59; 1.52)	0.62 * (0.41; 0.93)	1.73 * (1.05; 2.87)	1.61 * (1.07; 2.42)
City with less than 100,000 citizens	1.57 (0.98; 2.50)	1.57 (0.96; 2.59)	0.64 (0.40; 1.02)	1.13 (0.74; 1.73)	0.63 (0.39; 1.05)	0.89 (0.58; 1.34)
**Self-assessed income** (*ref. high*)						
Low	1.31 (0.73; 2.35)	2.55 ** (1.38; 4.69)	0.76 (0.43; 1.37)	1.42 (0.82; 2.48)	0.39 ** (0.21; 0.72)	0.70 (0.40; 1.23)
Average	0.83 (0.52; 1.35)	1.51 (0.92; 2.48)	1.20 (0.74; 1.94)	1.32 (0.88; 1.98)	0.66 (0.40; 1.09)	0.76 (0.51; 1.14)
**Importance of addition of grain and/or use of wholegrain flour** (*ref. 3rd tertile—unimportant attribute*)						
An important attribute (1st tertile)	0.68 (0.42; 1.09)	0.37 *** (0.22; 0.63)	1.47 (0.92; 2.36)	0.51 ** (0.32; 0.79)	2.67 *** (1.59; 4.48)	1.98 ** (1.27; 3.07)
A attribute of limited importance (2nd tertile)	0.74 (0.48; 1.14)	0.65 (0.40; 1.07)	1.35 (0.87; 2.08)	0.88 (0.56; 1.36)	1.53 (0.94; 2.51)	1.13 (0.74; 1.74)
**Importance of fiber addition** (*ref. 3rd tertile—unimportant attribute*)						
An important attribute (1st tertile)	0.74 (0.47; 1.17)	0.54 * (0.33; 0.87)	1.35 (0.86; 2.12)	0.69 (0.46; 1.03)	1.85 * (1.14; 3.00)	1.46 (0.97; 2.18)
A attribute of limited importance (2nd tertile)	1.20 (0.75; 1.91)	0.86 (0.52; 1.41)	0.84 (0.53; 1.33)	0.81 (0.52; 1.27)	1.17 (0.71; 1.93)	1.23 (0.79; 1.92)
**Interest in fiber content in a diet** (*ref. high*)						
Low	1.31 (0.87; 1.98)	1.73 * (1.10; 2.72)	0.76 (0.51; 1.15)	1.83 ** (1.23; 2.73)	0.58 * (0.37; 0.91)	0.55 ** (0.37; 0.81)
**Perception of roll as a source of fiber**						
A roll considered as containing the most fiber	10.52 *** (6.30; 17.59)	3.78 *** (2.10; 6.81)	0.10 *** (0.06; 1.16)	0.44 ** (0.24; 0.81)	4.50 *** (2.78; 7.28)	7.78 *** (5.32; 11.38)
**Checking the content of fiber on food labels** (*ref. not checking*)						
Checking	0.87 (0.51; 1.50)	1.29 (0.74; 2.23)	1.15 (0.67; 1.96)	1.52 (0.98; 2.34)	0.78 (0.45; 1.35)	0.66 (0.43; 1.02)

e^β^—OR, point estimate; 95% Cl—95% Wald Confidence Limits; Statistically significant: * *p* < 0.05, ** *p* < 0.01, *** *p* < 0.001 (Wald’s test).

## References

[1] King D.E., Mainous A.G., Lambourne C.A. (2012). Trends in Dietary Fiber Intake in the United States, 1999–2008. J. Acad. Nutr. Diet..

[2] Mobley A.R., Jones J.M., Rodriguez J., Slavin J., Zelman K.M. (2014). Identifying practical solutions to meet America’s fiber needs: Proceedings from the food & fiber summit. Nutrients.

[3] Stephen A.M., Champ M.M.J., Cloran S.J., Fleith M., Van Lieshout L., Mejborn H., Burley V.J. (2017). Dietary fibre in Europe: Current state of knowledge on definitions, sources, recommendations, intakes and relationships to health. Nutr. Res. Rev..

[4] Mann K.D., Pearce M.S., McKevith B., Thielecke F., Seal C.J. (2015). Low whole grain intake in the UK: Results from the National Diet and Nutrition Survey rolling programme 2008–2011. Br. J. Nutr..

[5] Thane C.W., Jones A.R., Stephen A.M., Seal C.J., Jebb S.A. (2005). Whole-grain intake of British young people aged 4–18 years. Br. J. Nutr..

[6] Gellynck X., Kuhne B., Van Bockstaele F., Van de Walle D., Dewettinck K. (2009). Consumer perception of bread quality. Appetite.

[7] Lambert J.L., Le-Bail A., Zuniga R., Van-Haesendonck I., Vnzeveren E., Petit C., Rosell M.C., Collar C., Curic D., Colic-Baric I. (2009). The attitudes of European consumers toward innovation in bread; interest of the consumers toward selected quality attributes. J. Sens. Stud..

[8] McKeown N.M., Jacques P.F., Seal C.J., de Vries J., Jonnalagadda S.S., Clemens R., Webb D., Murphy L.A., van Klinken J.-W., Topping D. (2013). Whole Grains and Health: From Theory to Practice—Highlights of the Grains for Health Foundation’s Whole Grains Summit 2012. J. Nutr..

[9] Monsivais P., Scarborough P., Lloyd T., Mizdrak A., Luben R., Mulligan A.A., Wareham N.J., Woodcock J. (2015). Greater accordance with the Dietary Approaches to Stop Hypertension dietary pattern is associated with lower diet-related greenhouse gas production but higher dietary costs in the United Kingdom. Am. J. Clin. Nutr..

[10] Baixauli R., Salvador A., Hough G., Fiszman S.M. (2008). How information about fibre (traditional and resistant starch) influences consumer acceptance of muffins. Food Qual. Prefer..

[11] Guiné R.P.F., Ferreira M., Correia P., Duarte J., Leal M., Rumbak I., Barić I.C., Komes D., Satalić Z., Sarić M.M. (2016). Knowledge about dietary fibre: A fibre study framework. Int. J. Food Sci. Nutr..

[12] Przeor M., Flaczyk E., Kmiecik D., Kobus-Cisowska J., Bueschke M., Kulczyński B. (2018). Polish Consumers’ Awareness and Knowledge About Functional Food. Folia Pomeranae Univ. Technol. Stetin. Agric. Aliment. Piscaria Zootech..

[13] Lyly M., Soini E., Rauramo U., Lähteenmäki L. (2004). Perceived role of fibre in a healthy diet among Finnish consumers. J. Hum. Nutr. Diet..

[14] Martin C., Chiron H., Issanchou S. (2013). Impact of dietary fiber enrichment on the sensory characteristics and acceptance of French baguettes. J. Food Qual..

[15] Ljubicic M., Saric M.M., Rumbak I., Baric I.C., Komes D., Satalic Z., Guiné R.P.F. (2017). Knowledge about dietary fibre and its health benefits: A cross-sectional survey of 2536 residents from across Croatia. Med. Hypotheses.

[16] Smith A.T., Kuznesof S., Richardson D.P., Seal C.J. (2003). Behavioural, attitudinal and dietary responses to the consumption of wholegrain foods. Proc. Nutr. Soc..

[17] Nicklas T.A., Jahns L., Bogle M.L., Chester D.N., Giovanni M., Klurfeld D.M., Laugero K., Liu Y., Lopez S., Tucker K.L. (2013). Barriers and Facilitators for Consumer Adherence to the Dietary Guidelines for Americans: The HEALTH Study. J. Acad. Nutr. Diet..

[18] Mialon V.S., Clark M.R., Leppard P.I., Cox D.N. (2002). The effect of dietary fibre information on consumer responses to breads and “English” muffins: A cross-cultural study. Food Qual. Prefer..

[19] Dewettinck K., Van Bockstaele F., Kuhne B., Van de Walle D., Courtens T.M., Gellynck X. (2008). Nutritional value of bread: Influence of processing, food interaction and consumer perception. J. Cereal Sci..

[20] Foschia M., Peressini D., Sensidoni A., Brennan C.S. (2013). The effects of dietary fibre addition on the quality of common cereal products. J. Cereal Sci..

[21] Ares G., Gambaro A. (2007). Influence of gender, age and motives underlying food choice on perceived healthiness and willingness to try functional foods. Appetite.

[22] Baixauli R., Sanz T., Salvador A., Fiszman S.M. (2008). Muffins with resistant starch: Baking performance in relation to the rheological properties of the batter. J. Cereal Sci..

[23] Crofton E.C., Markey A., Scannell A.G.M. (2013). Consumers’ expectations and needs towards healthy cereal based snacks: An exploratory study among Irish adults. Br. Food J..

[24] Tarrega A., Quiles A., Morell P., Fiszman S., Hernando I. (2017). Importance of consumer perceptions in fiber-enriched food products. A case study with sponge cakes. Food Funct..

[25] Feili R., Zzaman W., Wan Abdullah W.N., Yang T.A. (2013). Physical and Sensory Analysis of High Fiber Bread Incorporated with Jackfruit Rind Flour. Food Sci. Technol..

[26] Carrillo E., Varela P., Fiszman S. (2012). Effects of food package information and sensory characteristics on the perception of healthiness and the acceptability of enriched biscuits. Food Res. Int..

[27] Ginon E., Lohéac Y., Martin C., Combris P., Issanchou S. (2009). Effect of fibre information on consumer willingness to pay for French baguettes. Food Qual. Prefer..

[28] Makhlouf S., Jones S., Ye S.H., Sancho-Madriz M., Burns-Whitmore B., Li Y.O. (2019). Effect of selected dietary fibre sources and addition levels on physical and cooking quality attributes of fibre-enhanced pasta. Food Qual. Saf..

[29] Bioproducts’, Innovative Technologies of Pro-Health Bakery Products and Pasta with Reduced Caloric Value. http://www.bioprodukty.sggw.pl/SitePages/Stronagłówna.aspx.

[30] Bauer J.J. (2014). Selection Errors of Random Route Samples. Sociol. Methods Res..

[31] Williams C.L., Felt-Gunderson P. (2013). Analysis of Average Daily Fiber Intake Among Ready-to-Eat Cereal Consumers: Role of Whole-Grain Cereals in Closing the Fiber Gap. Am. J. Lifestyle Med..

[32] Martinho C., Correia A., Goncalves F., Abrantes J., Carvalho R., Guine R. (2013). Study About the Knowledge and Attitudes of the Portuguese Population About Food Fibres. Curr. Nutr. Food Sci..

[33] Lemmens S.G., Born J.M., Rutters F., Schoffelen P.F., Wouters L., Westerterp-Plantenga M.S. (2010). Dietary restraint and control over wanting following consumption of forbidden food. Obesity.

[34] Provencher V., Polivy J., Herman C.P. (2009). Perceived healthiness of food. If it’s healthy, you can eat more!. Appetite.

[35] Arvola A., Lähteenmäki L., Dean M., Vassallo M., Winkelmann M., Claupein E., Saba A., Shepherd R. (2007). Consumers’ beliefs about whole and refined grain products in the UK, Italy and Finland. J. Cereal Sci..

[36] Dean M., Shepherd R., Arvola A., Vassallo M., Winkelmann M., Claupein E., Lähteenmäki L., Raats M.M., Saba A. (2007). Consumer perceptions of healthy cereal products and production methods. J. Cereal Sci..

[37] Sun Y.C. (2008). Health concern, food choice motives, and attitudes toward healthy eating: The mediating role of food choice motives. Appetite.

[38] Crucean D., Debucquet G., Rannou C., le-Bail A., le-Bail P. (2019). Vitamin B4 as a salt substitute in bread: A challenging and successful new strategy. Sensory perception and acceptability by French consumers. Appetite.

[39] Quagliani D., Felt-Gunderson P. (2017). Closing America’s Fiber Intake Gap: Communication Strategies From a Food and Fiber Summit. Am. J. Lifestyle Med..

[40] Hellyer N.E., Fraser I., Haddock-Fraser J. (2012). Food choice, health information and functional ingredients: An experimental auction employing bread. Food Policy.

[41] Hornick B., Dolven C., Liska D. (2012). The fiber deficit, part II: Consumer misperceptions about whole grains and fiber: A call for improving whole-grain labeling and education. Nutr. Today.

[42] Campos S., Doxey J., Hammond D. (2011). Nutrition labels on pre-packaged foods: A systematic review. Public Health Nutr..

[43] Hearty Á.P., McCarthy S.N., Kearney J.M., Gibney M.J. (2007). Relationship between attitudes towards healthy eating and dietary behaviour, lifestyle and demographic factors in a representative sample of Irish adults. Appetite.

[44] van Kooten M., de Ridder D., Vollebergh W., van Dorsselaer S. (2007). What’s so special about eating? Examining unhealthy diet of adolescents in the context of other health-related behaviours and emotional distress. Appetite.

[45] Grujic S., Grujic R., Petrovic D., Gajic J. (2013). The Importance of Consumers’ Knowledge About Food Quality, Labeling and Safety in Food Choice. J. Food Res..

[46] Hoefkens C., Valli V., Mazzocchi M., Traill W.B., Verbeke W. (2013). European consumers’ perceived seriousness of their eating habits relative to other personal health risks. Prev. Med..

[47] Vereecken C.A., Keukelier E., Maes L. (2004). Influence of mother’s educational level on food parenting practices and food habits of young children. Appetite.

[48] Wronka I., Suliga E., Pawlińska-Chmara R. (2013). Perceived and desired body weight among female university students in relation to BMI-based weight status and socio-economic factors. Ann. Agric. Environ. Med..

